# Longer small bowel segments are resected in emergency surgery for ileocaecal Crohn’s disease with a higher ileostomy and complication rate

**DOI:** 10.1007/s10151-019-02104-9

**Published:** 2019-10-29

**Authors:** V. Celentano, D. P. O’Leary, A. Caiazzo, K. G. Flashman, F. Sagias, J. Conti, A. Senapati, J. Khan

**Affiliations:** 1 Colorectal Unit, Queen Alexandra Hospital, Portsmouth Hospitals NHS Trust, Portsmouth, UK; 2 University of Portsmouth, Portsmouth, UK; 3 University of Campania “Luigi Vanvitelli”, Naples, Italy

**Keywords:** Crohn’s disease, Laparoscopic colorectal surgery, Ileocaecal resection, Inflammatory bowel disease, Emergency surgery

## Abstract

**Background:**

Repeated intestinal resections may have disabling consequences in patients with Crohn’s disease even in the absence of short bowel syndrome. Our aim was to evaluate the length of resected small bowel in patients undergoing elective and emergency surgery for ileocolic Crohn’s disease.

**Methods:**

A prospective observational study was conducted on patients undergoing surgery for ileocolonic Crohn’s disease in a single colorectal centre from May 2010 to April 2018. The following patients were included: (1) patients with first presentation of ileocaecal Crohn’s disease undergoing elective surgery; (2) patients with ileocaecal Crohn’s disease undergoing emergency surgery; (3) patients with recurrent Crohn’s disease of the distal ileum undergoing elective surgery. The primary outcomes were length of resected small bowel and the ileostomy rate. Operating time, complications and readmissions within 30 days were the secondary outcomes.

**Results:**

One hundred and sixty-eight patients were included: 87 patients in the elective primary surgery group, 50 patients in the emergency surgery group and 31 in the elective redo surgery group. Eleven patients (22%) in the emergency surgery group had an ileostomy compared to 10 (11.5%) in the elective surgery group (*p* < 0.0001). In the emergency surgery group the median length of the resected small bowel was 10 cm longer than into the group having elective surgery for primary Crohn’s disease.

**Conclusions:**

Patients undergoing emergency surgery for Crohn’s disease have a higher rate of stoma formation and 30-day complications. Laparoscopic surgery in the emergency setting has a higher conversion rate and involves resection of longer segments of small bowel.

## Introduction

Over 80% of patients diagnosed with primary ileocolic Crohn’s disease (CD), who are typically young adults, have a surgical resection within 10 years of their diagnosis [1]. Of these, 30–50% will have symptomatic recurrence of disease during the first 5 years and 50–80% by 10 years after surgery [2]. Almost 40–50% of patients undergoing surgery for CD are likely to need further operations within 10–15 years [3], with smoking, a penetrating phenotype and previous small bowel surgery increasing the likelihood of postoperative recurrence.

Short bowel syndrome is a rare sequela of repeated surgical resection for CD, with a cumulative risk of home parenteral nutrition of 1.5% at 20 years after diagnosis [4]. Nevertheless, repeated intestinal resections leading to impaired gastrointestinal functioning with nutritional and vitamin deficiencies may have disabling consequences for the patients even in the absence of short bowel syndrome. Bile salt diarrhoea may occur in up to half of patients with CD following bowel resection [5] and loss of the ileocecal valve may increase the risk of small bowel bacterial overgrowth [6].

Surgery does not cure CD, and should be used restrictively, although it should not be regarded as the last resort [7]. Specific indications for surgery include abscesses, complex perianal or internal fistulae that are unresponsive or insufficiently responsive to medical therapy, fibrostenotic strictures with symptoms of partial or complete bowel obstruction, high-grade dysplasia, and cancer [8]. The majority of patients are treated with elective surgery; however, patients with intestinal perforation, peritonitis, excessive bleeding or toxic megacolon require urgent surgery. The risk of major abdominal surgery within the first 5 years of diagnosis has declined due to the advances in medical management of CD with the use of disease-modifying agents [9], unfortunately without this reflecting in shorter segments of bowel being resected when surgery occurs [10]. More importantly, inappropriate delay in surgery may increase surgical morbidity [11] and affect short- and long-term outcomes.

Aim of this prospective study is to evaluate the length of resected small bowel in patients undergoing elective and emergency surgery for ileocaecal CD, and to assess the ileostomy rate and postoperative complications in these two groups.

## Materials and methods

### Study design

All patients undergoing surgery for ileocolic CD from May 1st 2010 to April 30th 2018 were included in this single-centre observational study designed according to the strengthening the reporting of observational studies in epidemiology (STROBE) checklist [12]. Patients were divided into three groups to compare the length of resected small bowel, the stoma rate and the short-term outcomes of ileocolic resection in: (1) patients with first presentation of ileocaecal CD undergoing elective surgery; (2) patients with ileocaecal CD undergoing emergency surgery; (3) patients with recurrent CD of the distal ileum undergoing elective surgery. Data on patients undergoing laparoscopic surgery were collected prospectively on a dedicated database with an intention to treat analysis. Patients undergoing surgery for recurrent disease were also included, while patients undergoing surgery for colonic or rectal disease only were excluded. Data for patients who underwent open surgery during the study period were retrospectively retrieved from review of medical notes and via the National Emergency Laparotomy Audit (NELA) database. Emergency surgery was defined as surgical resection during the same unplanned hospital admission for an acute presentation with small bowel obstruction, peritonitis or intra-abdominal sepsis due to complications of CD.

The indication for surgical resection was discussed at a dedicated inflammatory bowel disease (IBD) multidisciplinary team meeting (MDT) involving gastroenterologists, colorectal surgeons, radiologists, pathologists, IBD and stoma nurses. Preoperative assessment included colonoscopy, magnetic resonance enterography and intestinal ultrasound.

### Primary and secondary outcomes

Length of resected small bowel and ileostomy rate were the primary outcomes. The secondary outcomes were operating time, length of hospital stay (LOS), complications, reoperations and rehospitalisation within 30 days, and were recorded prospectively.

### Data collection

Preoperative, operative and postoperative data were recorded for each patient. Preoperative parameters included age, sex, body mass index (BMI), comorbidities, American Society of Anesthesiologists (ASA) status, albumin and haemoglobin concentration, smoking status, weight loss, indication for surgery and preoperative medical therapy, Montreal classification.

Operative data included operating time, intraoperative complications, estimated operative blood loss, conversion rate, reason for conversion and use of temporary ileostomy. Length of resected small bowel was detailed in the histopathology report. Postoperative data included LOS, time to tolerate oral fluids and oral diet, time to resolution of ileus and postoperative complications according to the Dindo–Clavien classification [13].

### Statistical analysis

Categorical variables are presented as frequency or percentage and were compared with the use of the Chi-square test or Fisher’s exact test, as appropriate. Continuous variables are presented as mean (± standard deviation) or median (first and third quartile) and were compared with the use of Student’s *t* test. The Mann–Whitney *U* test was used for continuous, not normally distributed outcomes. Because of possible confounders (i.e. anti-tumour necrosis factor (TNF) use, use of steroids, preoperative weight loss) the primary outcomes were also evaluated in a multivariate analysis. Statistical analysis was performed by using the Statistical Package for Social Sciences (SPSS version 16.0; SPSS, Chicago, IL, USA) and GraphPad Prism version 8.0.2 for Windows (GraphPad Software, La Jolla, CA, USA, www.graphpad.com). All reported *p* values were two-tailed, and *p* values of less than 0.05 were considered to indicate statistical significance.

### Ethics

The study was conducted in accordance with the principles of the Declaration of Helsinki and ‘good clinical practice’ guidelines. The database was approved by the local ethics committee and informed consent was obtained from all patients.

## Results

### Patient characteristics

One hundred and sixty-eight patients were included: 87 patients in the elective primary surgery group, 50 patients in the emergency surgery group and 31 in the elective redo surgery group. In the elective primary surgery group 39% of the patients were male and the median age was 32 years (range 26–48 years), while in the emergency group 42% were male and the median age was 50 years (range 33–65 years) and in the elective redo surgery group 64% were male and median age was 46 years (range 35–59 years). None of the patients in the emergency surgery group had any previous CD resection.

Baseline patients’ characteristics are detailed in Table 1. Patients undergoing redo surgery for recurrent CD were more than 10 years older than patients undergoing primary resection, as expected, and more male patients underwent redo surgery compared to primary and emergency surgery. The rate of penetrating disease was similar in the emergency surgery and primary elective groups.

**Table 1:** Baseline patient characteristics

	Elective primary (*n* = 87)	Emergency (*n* = 50)	Elective redo (*n* = 31)
Age (years)	32.5 (26–48)	50.5 (33.7–65.2)*	46.5 (35–59)*
Male-to-female ratio	34:53	21:29	20:11 *
ASA class			
I	5	0	4
II	55	23	16
III	9	10	7
Missing data	18	17	4
BMI	23.75 (20–29.1)	26 (20.5–32.5)	25 (21.7–28)
Weight loss^†^	21 (24.1%)	10 (20%)	5 (16.1%)
Previous surgery^††^	16 (18.3%)	13 (26%)	31 (100%)*
Penetrating disease	15 (17.2%)	11 (22%)	2 (6.4%)*
Preoperative anti-TNF	41 (47.1%)	21 (42%)	7 (22.5%)*
Preoperative steroids^†††^	15 (17.2%)	14 (28%)	1 (3.2%)*

Data are expressed as number (percentage) and median (lower–upper quartile)

*BMI* body mass index, *ASA* American Society of Anesthesiologists, *TNF* tumour necrosis factor

*Statistically significant with *p* value < 0.0001

^†^Preoperative weight loss of > 5% during last 6 months

^††^Previous abdominal surgery

^†††^20 mg or more

### Ileostomy rate and length of resected small bowel

Patients undergoing emergency surgery were more likely to have a stoma fashioned at the time of the surgery. Eleven patients (22%) had an ileostomy in the emergency surgery group compared to 10 (11.5%) in the elective surgery group (*p* < 0.0001). At 24 months after surgery one patient in the emergency surgery group (2%) and two patients (2.3%) in the elective surgery group still had a stoma.

Emergency surgery for ileocolic CD carries a risk of a longer length of small bowel being resected (median length of resected small bowel 30.4 cm), with additional 10 cm of small bowel being resected compared to elective surgery for primary CD (median length of resected small bowel 19 cm, *p* < 0.0001). Significantly shorter segments of small bowel were resected in elective surgery performed for recurrent ileocolic CD (Table 2).

**Table 2:** Short-term outcomes for emergency, recurrent and elective ileocolic resection for CD

	Elective primary (*n* = 87)	Emergency (*n* = 50)	Elective redo (*n* = 31)
Open surgery	2 (2.3%)	15 (30%)*	1 (3.2%)
Laparoscopic surgery	85 (97.7%)	35 (70%)*	30 (96.8%)
Conversion to open	3 (3.5%)	4 (11.4%)*	4 (12.9%)*
Operating time (min)	140 (105–180)	170 (117.5–205)*	180 (143.8–198.8)*
Ileostomy formation	10 (11.5%)	11 (22%)*	2 (6.4%)
LOS (days)	6 (5–8)	8 (5–13)*	6 (4.7–10)
Readmissions	11 (12.6%)	5 (10%)	6 (19.3%)
Reoperations	1 (1.1%)	2 (4%)*	2 (6.4%)*
30-day complications	20 (22.9%)	17 (34%)*	9 (29%)
Length of resected small bowel (cm)	19 (13–26)	30.4 (20–42)*	11 (8–17)

Data are expressed as number (percentage) and median (lower–upper quartile)

*LOS* length of hospital stay

*Statistically significant with *p* value < 0.0001

The length of resected small bowel was longer than 40 cm in 14 patients (28%) in the emergency surgery group, compared to 7 (8%) and none in the elective surgery groups for primary and recurrent CD, respectively (*p* < 0.0001).

### Operating time, conversion rate and LOS

Conversion rate was 11.4% in the emergency surgery group, similar to 12.9% in the elective redo surgery group, but significantly different from 2.4% in the elective surgery group (*p* < 0.0001). Operating time was 30 min longer and LOS was 2 days longer in patients undergoing emergency surgery compared to elective primary ileocolic CD surgery (Table 2).

### 30-day morbidity and mortality

Seventeen patients (34%) in the emergency surgery group, 9 in the elective redo surgery group (29%), and 20 (22.9%) in the elective primary surgery group experienced complications (*p* < 0.0001). Complications are detailed in Table 3. There was no mortality.

**Table 3:** Detailed 30-day morbidity and reoperations

Elective surgery for primary ileocolic CD: 20 patients (22.9%) experienced a total of 26 complications
8 Wound infection 7 Ileus requiring total parenteral nutrition 4 Intra-abdominal collection treated with radiological guided drainage 3 Bleeding: 2 requiring transfusions, 1 treated with laparotomy and washout 2 Anastomotic leak: 1 treated conservatively with antibiotics, 1 requiring laparotomy and stoma formation 1 High-output stoma 1 Mechanical bowel obstruction due to internal hernia requiring laparotomy
Emergency surgery: 17 patients (34%) experienced a total of 22 complications
6 Intra-abdominal collection requiring radiological guided drainage 3 Mechanical bowel obstruction: two treated conservatively, one requiring reoperation 3 Wound infection 3 High-output stoma 2 Anastomotic leak: 1 treated conservatively with antibiotics, 1 treated with laparotomy and stoma formation 2 Ileus requiring total parenteral nutrition 1 Bleeding requiring transfusions 1 Enterocutaneous fistula 1 Parastomal hernia
Elective redo surgery for recurrent ileocolic CD: 9 patients (29%) experienced a total of 12 complications
6 Wound infection 3 Ileus requiring total parenteral nutrition 2 Anastomotic leak requiring laparotomy and stoma formation 1 Bleeding requiring transfusions

*CD* Crohn’s disease

### Readmissions and reoperations

Five patients (10%) in the emergency surgery group were readmitted within 30 days from discharge, compared to 6 (19.3%) and 11 (12.6%) in the redo surgery and primary elective surgery groups, respectively (*p* < 0.0001). The reoperation rate did not significantly differ amongst the three groups. Reasons for reoperations are detailed in Table 3.

### Multivariate analysis

Multivariate analysis was performed to test for potential confounding factors in view of the small sample size. Length of resected small bowel and ileostomy rate in the emergency and elective groups were evaluated in a multivariable logistic regression model, with no statistically significant difference demonstrated regarding age (*p* = 0.42), preoperative weight loss > 5% (*p* = 0.54), ASA grade (*p* = 0.88), BMI (*p* = 0.31), preoperative steroids > 20 mg (*p* = 0.8) and anti-TNF treatment (*p* = 0.68).

## Discussion

Emergency surgery for primary ileocolic CD carries a risk of longer segments of small bowel being resected and a higher likelihood of an ileostomy being fashioned at the time of the surgery, compared to elective surgery for primary ileocolic CD. A median length of 23 cm of resected small bowel has been recently reported in patients undergoing surgery for CD, with a cumulative length of resected small bowel of 36 cm at 15 years [14]. Our study has similar findings, with a median of 30 and 19 cm of resected ileum in the emergency and primary elective surgery groups, respectively.

It is important to note that the risk of short bowel syndrome in CD is low [15] and is mainly due to repeated operations because of complications, rather than recurrence, and that the length of remaining small bowel is more important than the extent of resection [16]. Nevertheless, multiple resections of the diseased bowel may result in functional diarrhoea, fat malabsorption, and ultimately short bowel syndrome, requiring parenteral nutrition treatment, and affecting patients’ quality of life with selective vitamin deficiencies and malnutrition. The Lémann index assesses globally the cumulative structural bowel damage that can occur in CD [17]. Surgical resection of the bowel, being irreversible, is considered the maximum level of bowel damage in this index. The significantly shorter length of resected small bowel in patients undergoing redo surgery for recurrent ileocolic CD has been previously demonstrated [18] and may be explained by the fibrostenotic phenotype that typically involves anastomotic CD recurrence [19] and for this reason we considered recurrent CD as a separate group in this study.

Prompt planning of elective surgery and high-volume IBD surgeons may impact on length of the resected specimen, stoma rate and postoperative outcomes when surgery is performed in specialist referral centres, which may be more often the case in recurrent CD, with surgeons more aware of the risk of short bowel syndrome and more familiar with the use of bowel-preserving techniques (i.e. strictureplasties) [20]. These hypotheses, however, need to be addressed in larger prospective studies. During the 9-year study period 50 patients (29.7%) within the total population of patients requiring surgery for CD at our institution, had an emergency operation, which is in agreement with previously reported rates of 35% [21] and 42% [22] for non-scheduled surgery in CD, highlighting the difficulties in predicting the course of disease in a significant proportion of patients. It is important to consider how these rates may be affected by the adopted denotation of emergency surgery, which was broadly defined in our study as surgical resection during the same unplanned hospital admission for an acute presentation of CD.

Our study reported a rate of stoma formation of 11.5% when surgery is performed electively, compared to 22% in the emergency setting. The presence of a stoma can significantly affect patients’ quality of life [23, 24] and is also associated with a risk of complications and re-interventions [25]. A stoma rate up to 35% has been described for complicated CD [26] and a penetrating phenotype of CD may explain a more common use of stomas in selected patients, because of abscesses and intra-abdominal contamination, or due to complex fistulae requiring more than one intestinal resection. Nevertheless, stricturing disease is the most common indication for surgery in ileocolic CD [27] and no difference was found in our study in the rate of penetrating phenotype between the emergency and elective surgery groups. A recent meta-analysis reported that a third of the ileostomies are reversed with a stapled technique, resulting in further small bowel resection [28], which needs to be taken into account when considering the cumulative bowel loss in patients undergoing emergency surgery and where the surgery is complicated by anastomotic leak requiring relaparotomy.

Our study found a high complication rate following ileocolic CD surgery, varying from 22.9 to 34% in elective and emergency surgery, similar to the published literature [29]. The association between procedural volume and surgical outcomes is well-described throughout all types of surgery, including those for IBD [30] with up to a twofold in-hospital mortality increase in low-volume hospitals [31]. Surgery for CD is technically challenging, due to multifocal disease, a thickened mesentery and the potential for fistulae, abscesses, and large phlegmons [32] and the perioperative decision-making of when to operate and whether to fashion an anastomosis or to create a stoma, requires highly trained surgeons [33]. Nevertheless, the underlying mechanisms for the relationship between surgical volume and postoperative mortality are likely multifactorial and more complex than just surgeon experience as higher volume hospitals may have more institution-level-related resources and infrastructure such as operating room volume and intensive care unit beds that may facilitate surgery. Patients with CD require a multidisciplinary approach [34] for an essential close and structured integration of medical and surgical management to identify the right time for surgery with the aim of preventing emergency surgery, postoperative complications and recurrence. It is a quality requirement that patients having surgery for IBD have it undertaken by a colorectal surgeon who is a core member of the IBD multidisciplinary team [35] auditing stoma rate, complications, re-interventions and mortality [36].

This is a single-centre study with patients being recruited within a study period of 9 years, and concerns about cases being performed at different stages of the learning curve might be raised, particularly with relation to conversion to open surgery. The cumulative conversion rate was 7.3% (11 cases out of 150), however 7 of the 11 conversions occurred in the last 3 years of the study period, suggesting more complex cases being approached laparoscopically rather than being due to the learning curve. Another limitation of our study is that no direct patient reported outcome measures were assessed and no data were collected on smoking status and rate and length of postoperative admission to intensive care amongst the three different groups of patients. Elective CD surgeries are all performed by dedicated colorectal surgeons in our department, while an emergency surgery subspecialty colorectal service provision was only introduced during the second half of the study period, introducing a bias on the contribution of training and expertise of the operating surgeons. Finally, the effect of emergency surgery on the risk of clinical and surgical recurrence cannot be evaluated because of the lack of long-term follow-up, and a larger prospective study, with patients stratified according to the risk of recurrence is desirable.

## Conclusions

Patients who have  emergency surgery for CD have a higher rate of stoma formation and 30-day complications. Laparoscopic surgery for CD in the emergency setting has a higher conversion rate and involves resection of longer segments of small bowel.
